# Early trends and predictors of renal function following computed tomography-guided percutaneous cryoablation of a renal mass in patients with and without prior renal impairment

**DOI:** 10.1590/0100-3984.2019.0098

**Published:** 2020

**Authors:** Pedro V. Staziaki, Harshna V. Vadvala, Vanessa Fiorini Furtado, Dania Daye, Ronald S. Arellano, Raul N. Uppot

**Affiliations:** 1 Massachusetts General Hospital, Harvard Medical School, Boston, MA, USA.; 2 Boston Medical Center, Boston University School of Medicine, Boston, MA, USA.; 3 Johns Hopkins Hospital, Johns Hopkins University, Baltimore, MD, USA.

**Keywords:** Radiology, interventional, Renal insufficiency, Kidney/physiopathology, Glomerular filtration rate, Tomography, X-ray computed/methods, Radiologia intervencionista, Insuficiência renal, Rim/fisiopatologia, Taxa de filtração glomerular, Tomografia computadorizada/métodos

## Abstract

**Objective:**

To assess trends and predictors of the glomerular filtration rate (GFR) after renal mass cryoablation in patients with and without history of renal impairment.

**Materials and Methods:**

This was a retrospective study of 39 patients who underwent computed tomography-guided percutaneous cryoablation of a renal mass, divided into two groups: those with prior renal impairment (PRI+); and those without prior renal impairment (PRI−). The GFR trend and the chronic kidney disease stage were evaluated at baseline, as well as at 1, 6, and 12 months after cryoablation. Predictors of GFR at 1 and 6 months were modeled with linear regression.

**Results:**

In both groups, the mean GFR at 1 month and 6 months was significantly lower than at baseline (*p* < 0.001 and *p* = 0.01, respectively). Although the GFR was lower across all time points in the PRI+ group (−26.1; *p* < 0.001), the overall trend was not statistically different from that observed in the PRI− group (*p* = 0.89). Univariate analysis showed that the decline in GFR at 1 and 6 months correlated with the baseline GFR (0.77 and 0.63; *p* < 0.001 and *p* = 0.03, respectively) and with the size of the ablation zone (−7.6 and −12.84, respectively; *p* = 0.03 for both). However, in the multivariate model, baseline GFR was predictive only of GFR at 1 month (*p* < 0.001).

**Conclusion:**

The trend in GFR decline after cryoablation is similar for patients with and without a history of renal impairment. Baseline GFR predicts the mean GFR in the early post-cryoablation period.

## Abstract (pt)

**Objetivo:**

Medir as tendências iniciais da função renal pela taxa de filtração glomerular (TFG) em pacientes com e sem comprometimento renal prévio após crioablação renal.

**Materiais e Métodos:**

Este é um estudo retrospectivo de 39 pacientes submetidos a crioablação percutânea guiada por tomografia computadorizada de massa renal. Os pacientes foram divididos em dois grupos: com comprometimento renal prévio (CRP+) e sem comprometimento renal prévio (CRP-). As tendências da TFG foram avaliadas nos tempos 0, 1, 6 e 12 meses com o estadiamento de doença renal crônica. Preditores da TFG em 1 e 6 meses foram modelados usando regressão linear.

**Resultados:**

Em ambos os grupos houve declínio da TFG média após 1 e 6 meses (*p* < 0,001 e *p* = 0,01, respectivamente). Apesar de o grupo CRP+ demonstrar média menor da TFG em cada um dos tempos (−26,1; *p* < 0,001), a tendência de forma geral não foi estatisticamente diferente do grupo CRP- (*p* = 0,89). Análise univariada mostrou correlação da TFG no tempo 0 (0,77 e 0,63; *p* < 0,001 e *p* = 0,03, respectivamente) e tamanho da ablação (−7,6 e −12,84; *p* = 0,03), com declínio em 1 e 6 meses. Porém, no modelo multivariado, apenas a TFG no tempo 0 foi preditiva da TFG em 1 mês (*p* < 0,001).

**Conclusão:**

A curva de tendência de declínio da TFG após crioablação foi similar entre os pacientes dos grupos CRP+ e CRP-, sem mudanças no estadiamento de função renal. A TFG no tempo 0 foi preditiva da TFG média no período de crioablação inicial.

## INTRODUCTION

The advent of cross-sectional imaging has made it possible to identify smaller renal masses^(1,2)^. Although nephrectomy has historically been the established standard of treatment for such masses, it has been shown to increase the risk of chronic kidney disease (CKD) and might therefore no longer be considered the gold-standard treatment for small renal tumors^(3)^. Even partial nephrectomy has been shown to increase the risk of a decline in renal function by up to 20%^(4)^. Therefore, minimally invasive technologies, also known as nephron-sparing approaches, are rapidly gaining acceptance.

Percutaneous cryoablation is considered for patients who present with small, incidentally detected renal tumors (ideally < 4 cm in diameter and limited to the kidney), as well as for those who are poor surgical candidates (because of advanced age or significant comorbidity) and those who need nephron-sparing treatment options as a way to postpone or avoid the need for chronic dialysis. The last category often includes patients with CKD, a single kidney, or a genetic predisposition to multiple tumors. In addition, it is vital to monitor renal function in the early post-procedure period, especially in cancer patients with comorbidities, because further exposure to nephrotoxic agents can complicate their recovery.

Cryoablation has many advantages over traditional surgery, such as having a lower incidence of serious complications^(5,6)^ and producing less morbidity in the immediate post-procedure period^(7)^, as well as being more affordable^(8)^. Initial studies of cryoablation demonstrated favorable outcomes^(9-11)^ and proved it to be a safe, effective treatment^(12-17)^. Nevertheless, renal function is one of the most important considerations in devising treatment strategies for the management of renal masses and it remains unclear to what extent cryoablation affects renal function, particularly among patients with a history of renal impairment.

The objective of this study was to determine trends in renal function after cryoablation of a renal mass in patients with and without a history of renal impairment, comparing the two groups. A secondary objective was to identify predictors of post-cryoablation renal function.

## MATERIALS AND METHODS

### Study design

This was a single-center retrospective study. This investigation was approved by the local institutional review board, which waived the need for informed consent. We included all consecutive patients who underwent computed tomography (CT)-guided percutaneous cryoablation of a renal mass at a tertiary-care teaching hospital between April 2009 and August 2012. Patients who did not undergo follow-up renal function testing were excluded, as were those subsequently requiring additional cryoablation treatment.

The eligible patients were divided into two groups: those with prior renal impairment (PRI+) and those without (PRI-). Patients were included in the PRI+ group if they had a glomerular filtration rate (GFR) < 60 mL/min/1.73 m^2^, a reported diagnosis of CKD, a congenital single kidney, or a history of radical or partial nephrectomy, as well as if they had previously undergone renal thermal ablation. The patients in the PRI− group had no history of renal impairment or renal intervention (i.e., had healthy kidneys).

The renal function marker of interest was the GFR, which was determined at four time points: baseline (the last measurement before ablation); and 1, 6, and 12 months after ablation. The primary endpoint was a post-cryoablation trend toward a decline in renal function. The secondary endpoints were declines in GFR at 1 month and 6 months after cryoablation.

### Variables of interest

Baseline and post-ablation renal function was assessed on the basis of the GFR, calculated with the Modification of Diet in Renal Disease formula^(18)^, which is based on serum creatinine levels, and was recorded as a continuous variable. Covariates with a known influence on renal function were recorded at admission for the cryoablation procedure. In brief, covariates included age, sex, race, hypertension, diabetes mellitus, dyslipidemia, body mass index (BMI), smoking history, and alcohol abuse.

### Statistical analysis

Baseline characteristics are presented as frequency (percentage) for categorical data and as mean ± standard deviation or median (interquartile range) for continuous data. Continuous data were compared by using unpaired Student’s t-tests or Wilcoxon rank-sum tests, as appropriate. Categorical data were compared by using the chi-square or Fisher’s exact test.

The trend in renal function was evaluated in a longitudinal analysis of measurements obtained at four time points. The mean response was modeled using analysis of response profiles and tested for parallelism between the two groups. If both trends were parallel, the means were tested for time effect and group effect.

Multivariate regression was used in order to identify independent predictors of renal function at 1 month and 6 months after cryoablation. In the univariate analysis, we assessed the association of known predictors of renal impairment, which included advanced age, male sex, African American race, diabetes, hypertension, dyslipidemia, high BMI, and smoking, as well as variables related to renal disease, such as baseline GFR, a history of radical nephrectomy, a history of partial nephrectomy, prior ablation, and the size of the tumor. In the multivariate analysis, we included variables with a *p* < 0.10 in the univariate analysis.

All statistical analyses were performed with the R software, version 3.2.2 (The R Foundation for Statistical Computing, Vienna, Austria). A two-sided *p*-value < 0.05 was considered statistically significant.

## RESULTS

### Patient characteristics

During the study period, 51 patients underwent CT-guided percutaneous cryoablation of a renal mass at our institution. Twelve patients were excluded because of a lack of follow-up renal function testing or because they subsequently required additional cryoablation. Therefore, the final sample comprised 39 patients, of whom 19 (49%) were in the PRI− group and 20 (51%) were in the PRI+ group. The characteristics of the patients are presented in Table 1.

**Table 1:** Patient characteristics.

		Prior renal impairment		
Variable	All patients (n = 39)	No (n = 19)	Yes (n = 20)	*P*-value
Age, mean ± SD	68 ± 11	66 ± 12	69 ± 11	0.36
Race, n (%)				
White	34 (87.0)	16 (84.0)	18 (90.0)	0.66
African American	4 (10.0)	2 (11.0)	2 (10.0)	1.00
Asian	1 (2.6)	1 (0.5)	0	0.48
Male sex, n (%)	30 (76.9)	14 (73.7)	16 (80.0)	0.70
BMI (kg/m^2^), mean ± SD	33 ± 7.9	33 ± 8.9	33 ± 6.9	0.98
Hypertension, n (%)	33 (84.6)	14 (73.7)	19 (95.0)	0.09
Diabetes, n (%)	14 (35.9)	8 (42.0)	6 (30.0)	0.50
Dyslipidemia, n (%)	22 (56.4)	8 (42.0)	14 (70.0)	0.08
Smoking, n (%)	24 (61.4)	12 (63.0)	12 (60.0)	0.59
Alcohol abuse, n (%)	13 (34.2)	6 (31.0)	7 (35.0)	1.00
Follow-up (days), median (IQR)	70 (23-203)	89 (20-250)	63 (30-165)	0.61
Baseline creatinine, mean ± SD	1.1 ± 0.4	0.9 ± 0.8	1.3 ± 0.4	< 0.001
Baseline GFR (mL/min/1.73 m^2^), mean ± SD	71 ± 25	85.5 ± 19	59.4 ± 23	< 0.001
Type of prior renal impairment, n (%)				
CKD	11 (44.0)	0	11 (55.0)	< 0.001
Single kidney	3 (7.7)	0	3 (15.0)	0.21
Radical nephrectomy	2 (5.1)	0	2 (10.0)	0.49
Partial nephrectomy	5 (12.8)	0	5 (25.0)	< 0.05
Prior renal ablation	7 (17.9)	0	7 (35.0)	< 0.01
Repeated renal ablation	1 (2.6)	0	1 (5.0)	0.02

SD, standard deviation; IQR, interquartile range.

### Tumor characteristics and ablation results

Details pertaining to the tumor characteristics and ablation results are presented in Table 2. Because some patients had more than one renal mass, there were 44 renal masses in total. Of those, 20 were in PRI− group patients and 24 were in PRI+ group patients. The mean tumor size was 3.1 ± 1.2 cm in the PRI− group and 2.8 ± 1.2 cm in the PRI+ group, the overall mean size being 3.0 ± 1.2 cm.

**Table 2:** Characteristics of renal tumors submitted to cryoablation.

		Prior renal impairment		
Variable	All patients (n = 39)	No (n = 19)	Yes (n = 20)	*P* -value
Number of lesions ablated	44	20	24	-
Number of probes	5	2	3	-
Laterality, n (%)				
Right	18 (46.1)	9 (47.0)	9 (45.0)	1.00
Left	20 (51.3)	10 (53.0)	10 (50.0)	1.00
Both	1 (2.5)	0	1 (5.0)	1.00
Tumor size (cm), mean ± SD	3.0 ± 1.2	3.1 ± 1.2	2.8 ± 1.2	0.46
Location in the kidney, n (%)				
Upper pole	14 (36.0)	8 (42.1)	6 (30.0)	0.53
Middle pole	7 (18.0)	2 (10.5)	5 (25.0)	0.41
Lower pole	14 (36.0)	8 (42.1)	6 (30.0)	0.51
Multiple poles	4 (10.0)	1 (5.3)	3 (15.0)	0.62
Subtype of renal cell carcinoma, n (%)				
Clear cell	24 (61.5)	12 (63.0)	12 (60.0)	1.00
Papillary	10 (26.0)	4 (21.0)	6 (30.0)	0.71
Chromophobe	1 (2.5)	0	1 (5.0)	1.00
Multiple	1 (2.5)	1 (5.3)	0	1.00
Unknown	1 (2.5)	1 (5.3)	0	1.00
Metastasis, n (%)	2 (5.0)	1 (5.3)	1 (5.0)	1.00

SD, standard deviation.

**Figure 1:**
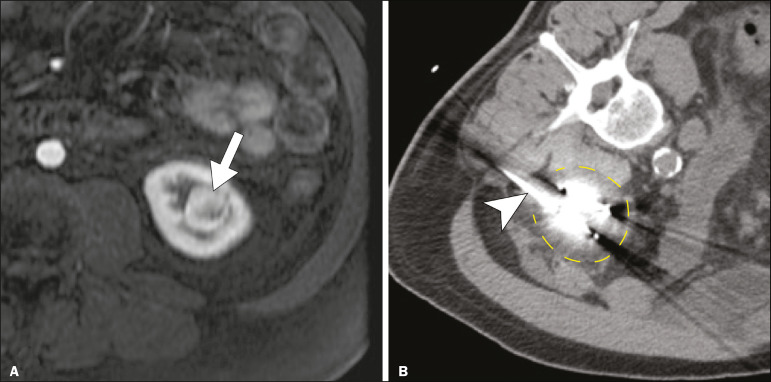
A 69-year-old male with a clear cell renal cell carcinoma measuring 2.5 cm. **A:** Pre-procedure magnetic resonance imaging scan, in the arterial phase, showing an arterially enhancing lesion (arrow) in the middle pole of the left kidney. **B:** CT-guided cryoablation of the same patient in the oblique left lateral decubitus position. One of the three cryoablation probes can be seen in the image (arrowhead), as can an ice ball forming around the probe tip (dashed yellow line).

In all cases, CT-guided percutaneous cryoablation of renal tumors was performed with a mean of three cryoprobes (range, 1-6), with a length of 15-17 cm and a width of 2.4 mm, and the freeze-thaw-freeze time was typically 10-8-10 min (Figure 1). There was no overall difference in the laterality of the lesions targeted for ablation, which were in the right kidney in 18 patients (46%), in the left kidney in 20 (51%), and in both kidneys in one (2.5%). Of the 39 ablated tumors, 37 (95%) were renal cell carcinomas, one of which is shown in Figure 1. The two remaining tumors were metastases. In one PRI+ group patient, three renal lesions were ablated concurrently.

### Renal function trends

**Figure 2:**
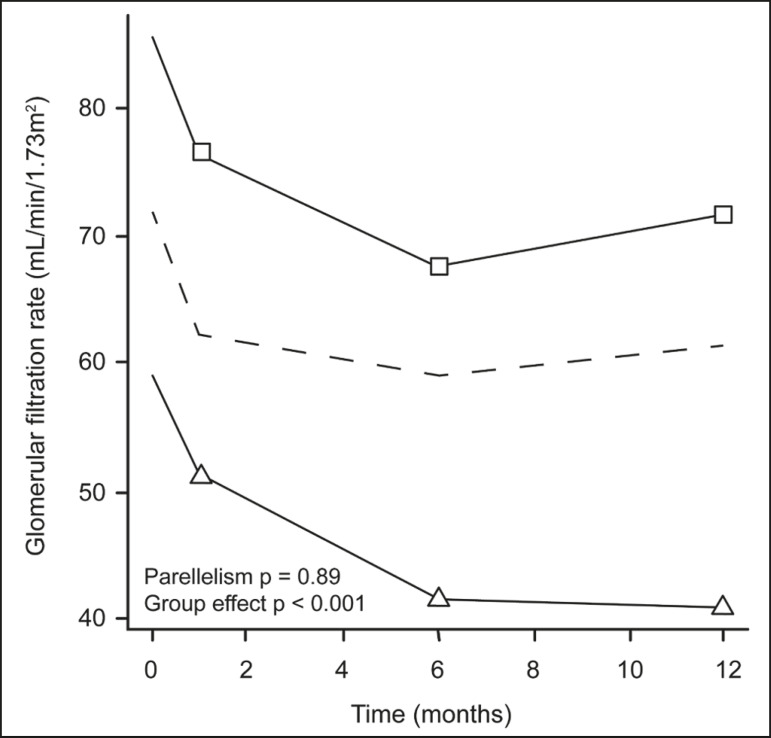
Mean GFR trend over 12 months in the PRI− group (squares), the PRI+ group (triangles), and the sample as a whole (dashed line). Despite the post-cryoablation decline in renal function, the GFR never dropped below 30 mL/min/1.73 m^2^ in either group.

The temporal trend in GFR (as a proxy for renal function) is plotted in Figure 2. The GFR values over the 12 months evaluated are shown in Table 3. Table 4 shows the mean and percent decline in GFR.

**Table 3:** GFR trends at multiple time points.

		Prior renal impairment		
Time point	All patients (n = 39)	No (n = 19)	Yes (n = 20)	*P*-value
Baseline	71 ± 25	85.5 ± 19	59.4 ± 23	< 0.001
1 month	62 ± 22	76.4 ± 19	51.3 ± 19	0.001
6 months	60 ± 20	67.6 ± 22	41.5 ± 5	0.01
12 months	61 ± 31	71.7 ± 33	40.7 ± 5	0.07

**Table 4:** Mean and percent GFR decline over 1 year.

		Prior renal impairment	
Time point	All patients (n = 39)	No (n = 19)	Yes (n = 20)
1 month	9 (-12.7)	9.1 (-10.6)	8.1 (-13.6)
6 months	11 (-15.5)	17.9 (-20.9)	17.9 (-30.1)
12 months	10 (-14)	13.8 (-16.1)	18.7 (-31.5)

Regarding the overall trend in our cohort, there was decline in the mean GFR at 1 month and 6 months after cryoablation, respectively, of −9.1% (95% confidence interval [95 % CI]: −15.9 to −2.1; *p* = 0.01) and −15.4% (95% CI: −28.1 to −2.6; *p* = 0.02). At 12 months after cryoablation, there was a slight recovery of renal function in the PRI− group (to −5.4%; 95% CI: −14.8 to 3.9), although the difference was not statistically significant (*p* = 0.25). In the analysis of the response profiles, the mean GFR was found to be lower, at all time points, in the PRI+ group (group estimate of −26.1; 95% CI: −39.3 to −12.8; *p* < 0.001), although testing for parallelism revealed that the trend curve was not statistically different (*p* = 0.89).

### Predictors of renal function

Table 5 shows the results of the univariate and multivariate analyses performed for the 1 month after cryoablation time point. In the univariate analysis, a decline in GFR at 1 month correlated positively with the baseline GFR (0.77; 95% CI: 0.59 to 0.95; *p* < 0.001) and negatively with the size of the ablation zone (−7.6; 95% CI: −14.5 to −0.7; *p* = 0.03). In the multivariate analysis, baseline GFR retained its effect (0.78; 95% CI: 0.6 to 1.0; *p* < 0.001), although the size of the ablation zone was no longer a significant predictor (0.34; 95% CI: −4.1 to 4.8; *p* = 0.88).

**Table 5:** Linear regression for the 1 month after cryoablation time point.

	Univariate analysis			Multivariate analysis	
Variable	Estimate	*P*-value		Estimate	*P*-value
Age	-0.5 (-1.2 to 0.2)	0.13		-	-
Male sex	-15.5 (-34 to 3.0)	0.10		-	-
African American	-15.5 (-43.6 to 12.6)	0.27		-	-
race					
Hypertension	-14.6 (-42.8 to 13.6)	0.30		-	-
Diabetes mellitus	1.1 (-17.7 to 19.9)	0.90		-	-
Dyslipidemia	3.3 (-14.2 to 20.9)	0.70		-	-
BMI	0.3 (-0.8 to 1.4)	0.58		-	-
Smoking	-1.4 (-18.8 to 16.0)	0.87		-	-
Baseline GFR	0.77 (0.59 to 0.95)	< 0.001		0.78 (0.6 to 1.0)	< 0.001
Prior radical	-15.8 (-50.6 to 18.9)	0.36		-	-
nephrectomy					
Prior partial	2.2 (-23.1 to 27.6)0	0.86		-	-
nephrectomy					
Single kidney	-21.7 (-49.2 to 5.8)	0.12		-	-
Prior ablation	-7.5 (-28.9 to 13.8)	0.48		-	-
Lesion size	-7.6 (-14.5 to -0.7)	0.03		0.34 (-4.1 to 4.8)	0.88

Table 6 shows the results of the univariate and multivariate analyses performed for the 6 months after cryoablation time point. In the univariate analysis, a decline in GFR at 6 months correlated positively with the baseline GFR (0.63; 95% CI: 0.1 to 1.2; *p* = 0.03) and negatively with the size of the ablation zone (−12.84; 95% CI: −23.8 to −1.9; *p* = 0.03). However, in the multivariate analysis, no significant effect was observed for the baseline GFR (0.41; 95% CI: −0.2 to 1.02; *p* = 0.16) or for the size of the ablation zone (−8.6; 95% CI: −20.8 to 3.6; *p* = 0.15).

**Table 6:** Linear regression for the 6 months after cryoablation time point.

	Univariate analysis			Multivariate analysis	
Variable	Estimate	*P*-value		Estimate	*P*-value
Age	-0.8 (-1.8 to 0.1)	0.08		-	-
Male sex	-15.5 (-18.1 to 17.2)	0.32		-	-
African American	6.3 (-33.4 to 46.1)	0.73		-	-
race					
Hypertension	-23.6 (-60.0 to 12.7)	0.18		-	-
Diabetes mellitus	21.1 (-10.0 to 52.1)	0.16		-	-
Dyslipidemia	10.8 (-18.0 to 39.6)	0.42		-	-
BMI	1.2 (-0.3 to 2.7)	0.1		-	-
Smoking	20.5 (-7.6 to 48.6)	0.13		-	-
Baseline GFR	0.63 (0.1 to 1.2)	0.03		0.41 (-0.2 to 1.02)	0.16
Prior radical	-12.0 (-68.6 to 44.6)	0.6		-	-
nephrectomy					
Single kidney	-10.9 (-64.2 to 42.5)	0.66		-	-
Prior ablation	-10.9 (-64.2 to 42.5)	0.66		-	-
Lesion size	-12.84 (-23.8 to -1.9)	0.03		-8.6 (-20.8 to 3.6)	0.15

## DISCUSSION

In our study, we evaluated the trends in renal function (GFR) after cryoablation of renal masses, comparing patients with and without a history of renal impairment. Although renal function decreased after cryoablation, the renal disease staging, according to the latest (2012) National Kidney Foundation Kidney Disease Outcomes Quality Initiative guidelines^(19)^, remained unchanged in both groups. At 12 months after cryoablation, the patients in the PRI− group remained in the same GFR category they presented at baseline: stage G2 (CKD stage 2). At that same time point, the GFR category worsened from G3a to G3b among the patients in the PRI+ group, although the CKD stage remained the same as it had been at baseline: CKD stage 3. In addition, the GFR never dropped below 30 mL/min/1.73m^2^ in either group and none of the patients in either group required renal replacement therapy such as dialysis or kidney transplantation.

Regarding the overall trend in our cohort, there were declines in the mean GFR at 1 month and 6 months after cryoablation. The slight improvement in GFR at 12 months in the PRI− group patients was likely due to the presence of healthy nephrons with better regenerative capacity in those patients. Our finding that the mean GFR was significantly lower at all time points among the PRI+ group patients is in keeping with the condition of a history of renal impairment. These findings corroborate those of a study in which patients were followed for 3 months after cryoablation or partial nephrectomy and showed that the post-procedure decline in absolute GFR was significantly greater after cryoablation than after partial nephrectomy, although the rate of CKD stage progression was similar between the two groups, indicating clinical preservation of renal function in both^(20)^.

Our data are complimentary to those in the literature on microwave ablation. For example, Zhou et al.^(21)^ found CT-guided percutaneous microwave ablation to be a safe procedure, post-ablation imaging showing a complete response in 42 (95%) of the 44 tumors ablated and renal function remaining stable at 1 month after ablation. In addition, Urabe et al.^(22)^ found that, among patients undergoing cryoablation, the decline in renal function at 6 months after the procedure was comparable between those with CKD and those without. Those authors found that renal function was significantly impaired in the early post-cryoablation period and did not recover in the 6 months thereafter. In the present study, we assessed renal function for an additional 6 months (total of 12 months of follow-up) and found that, although it was not statistically significant, there was partial recovery of renal function at 12 months among patients without a history of renal injury. A number of studies have investigated the effect that ablation has on renal function in comparison with that of partial nephrectomy, and the results have been mixed^(23-27)^. The rate of renal function decline in our sample is comparable to the approximately 20% previously reported for patients who have recently undergone partial nephrectomy^(4)^.

Tumor size has been shown to be significantly associated with postoperative renal function, larger tumors increasing the risk of a decline^(28)^. However, it is difficult to determine whether tumor size or baseline GFR is a better individual predictor of postoperative renal function, because they are probably collinear covariates or intermediate variables, i.e., a large lesion may cause the baseline GFR to be lower, which would, in turn, cause the GFR at 6 months to be lower. Our findings resonate with those of Urabe et al.^(22)^, who showed that the percent decline in GFR on postoperative day 1 was the only independent predictor of GFR at 6 months after percutaneous cryoablation of renal tumors. Some of the variables we evaluated as predictors of post-cryoablation renal function are in keeping with those previously evaluated in the literature. Tumor size and location have been described as the most important factors determining whether renal cell carcinomas can be successfully treated^(12,29)^. In addition, the age-related decline in GFR has been shown to be significantly greater in males than in females, who are thought to be protected by estrogen^(30)^.

Our study has limitations. First, it was a retrospective study involving a small cohort. In addition, the follow-up period was relatively short. However, the goal of the study was to evaluate trends in and predictors of renal function in the early post-procedure period. Furthermore, there was no external control group of patients undergoing a different treatment for renal tumors. There is a need for additional studies with larger patient samples and longer follow-up periods in order to evaluate long-term outcomes.

## CONCLUSION

In the present study, the trend of post-cryoablation renal function decline was comparable between those with a history of renal impairment and those without. In addition, there was no change in CKD staging after cryoablation, in either group. Although baseline GFR predicts the GFR in the early post-cryoablation period, a review of the factors associated with renal function preservation may improve pre-procedure patient counseling and allow decisions regarding management to be individualized in patients undergoing cryoablation of renal masses.
